# Trifluoromethanesulfonamide vs. Non-Fluorinated Sulfonamides in Oxidative Sulfamidation of the C=C Bond: An In Silico Study

**DOI:** 10.3390/molecules25214877

**Published:** 2020-10-22

**Authors:** Anton V. Kuzmin, Mikhail Yu. Moskalik, Bagrat A. Shainyan

**Affiliations:** Siberian Division of Russian Academy of Sciences, A.E. Favorsky Irkutsk Institute of Chemistry, 664033 Irkutsk, Russia; kuzmin@lin.irk.ru (A.V.K.); moskalik@irioch.irk.ru (M.Y.M.)

**Keywords:** alkenes, trifluoromethanesulfonamide, non-fluorinated sulfonamides, oxidative sulfamidation, theoretical analysis

## Abstract

A theoretical analysis of the reaction of oxidative sulfamidation of several alkenes was performed in order to explain the various experimental observations and different reactivity of triflamide and non-fluorinated sulfonamides. Transformations occurring in the system alkene–sulfonamide in the presence of oxidative system (Bu*^t^*OCl + NaI) were analyzed at the MP2/DGDZVP//B3LYP/DGDZVP level of theory using the IEF-PCM method for taking into account the solvent acetonitrile (MeCN) effect. As the model substrates, styrene, trimethyl(vinyl)silane, dimethyl(divinyl)silane and diphenyl(divinyl)silane were chosen and mesylamide, triflamide, tosylamide and *p*-nosylamide were taken as the reagents. Bu*^t^*OI generated from Bu*^t^*OCl and NaI reacts with sulfonamides to give *N*-iodinated sulfonamides RSO_2_NHI and RSO_2_NI_2_ as active intermediates, the iodinating activity of the latter being notably higher. The analysis allowed to answer such challenging questions as different reactivity of nonfluorinated sulfonamides leading to aziridination and of triflamide resulting in the formation the main products of bis-triflamidation, or different regioselectivity of halogenation of styrene and trimethyl(vinyl)silane caused by a linear intermediate iodonium cation in the former case and a cyclic one in the latter.

## 1. Introduction

Reactions of sulfamidation and aziridination of alkenes are atom economy and green routes to C–N coupling reactions [1] widely used in organic synthesis [2,3], resulting in many of biologically active compounds [4,5,6]. Amides are able to react with non-activated alkenes only under harsh conditions [7]; therefore, many attempts were made to discover amidation/aziridination techniques under mild conditions as well as to increase the yield of the target products. At present, there are several approaches for the activation of sulfonamide, in which the sulfonamido-containing precursors are represented by λ^3^-iodinanes (PhI=NTs) [8,9,10,11,12,13,14] and -brominanes [15], azides [16,17,18], chloramine-T [19,20,21], bromamine-T [22,23], *N*,*N*-dichlorosulfonamides [24,25], *N*-bromosuccinimide (NBS) and *N*-iodosuccinimide (NIS) [26], nitrido complexes [27,28,29,30] and combinations of sulfonamides with external oxidants [31,32,33,34].

The use of chloramines RSO_2_NClNa allows to perform amination of unsaturated substrates without metal complex catalysts, whereas aziridination of alkenes and dienes is catalyzed, e.g., by N-bromoamides [35]. TsNIK is a more effective catalyst for aziridination of 4-methylstyrene with chloramine-T (yield 75%) than TsNBrNa (60%) or TsNClNa (45%) due to a weaker N–I bond in TsNIK favoring the formation of the complex of nitrenoid TsNI with Cu salts [36]. The reaction of olefins, chloramine-T and CO_2_ in the presence of ammonium bromide salts directly affords 5-substituted 2-oxazolidinones [37]. In the presence of transition metal salts, chloramines are excellent hydroxyaminating reagents. Thus, osmium salt-catalyzed reactions of alkenes with chloramine-T result in β-aminoalcohols—important substrates in the synthesis of natural and pharmaceutically valuable compounds [38]. CO_2_-induced haloamination of olefins with chlor- [39] and bromamines [40] was reported. The same reagents allow to prepare heterocycles of various size with high diastereoselectivity by intramolecular cyclization of γ-iodoolefins [41].

Dihalogenoamines RSO_2_NX_2_ are effective haloaminating reagents for activated alkenes, which do not need additional oxidants, but, unlike similar reactions of chloramines, require, as a rule, the presence of metal salt catalysts [42,43,44]. Dichloroamine-T reacts with α,β-unsaturated ketones in MeCN to give the Ritter-type products, which, under the action of KOH or K_2_CO_3_, give imidazolidines [45,46,47] or, with Et_3_N as the base and CuOTf, aziridines [48].

**Scheme 1:**
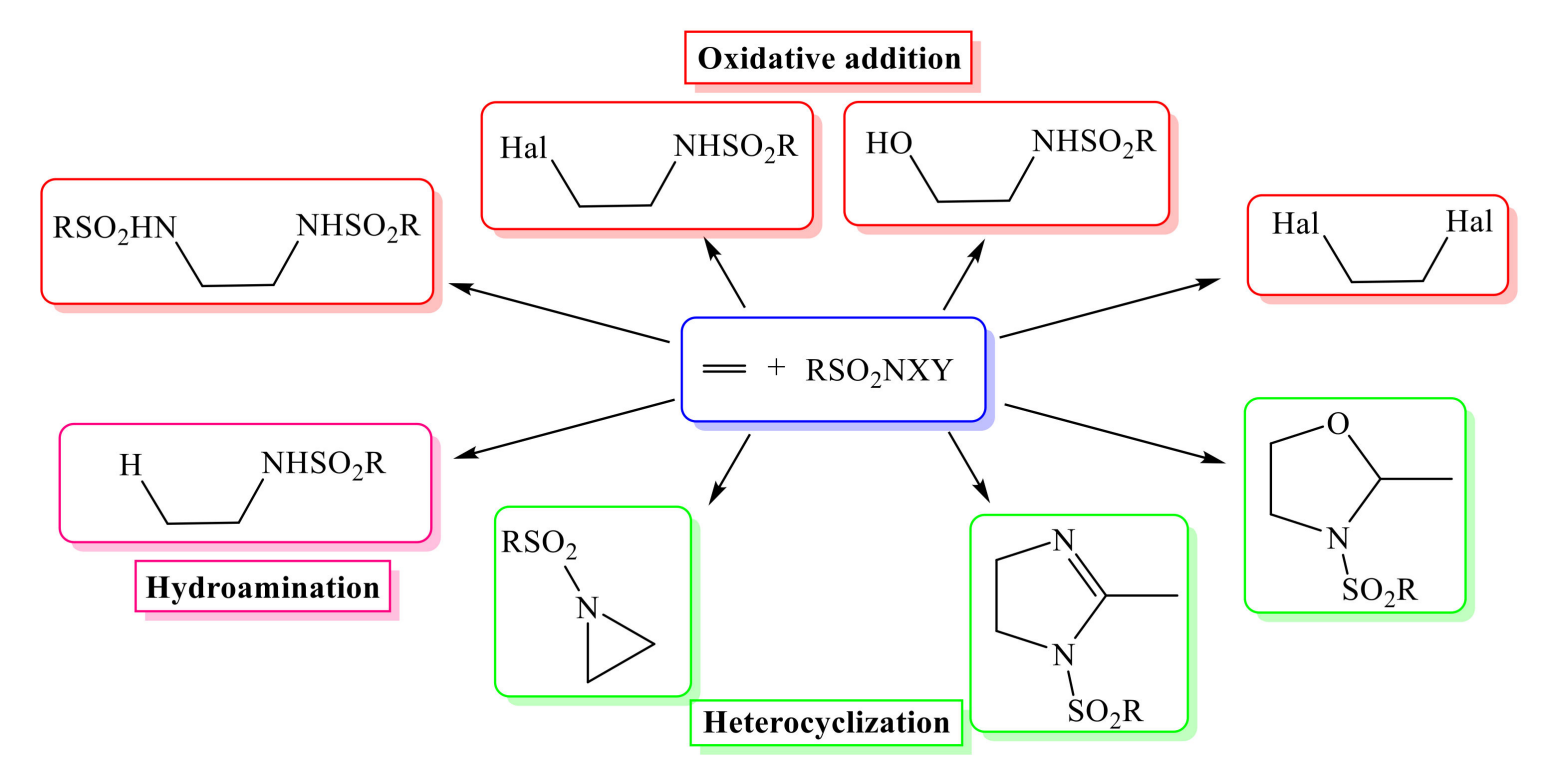
Versatile reaction pathways of alkenes with sulfonamide derivatives.

Iodinanes ArI=NSO_2_Ar’ are used mainly for aziridination of alkenes [9,49,50,51]. Note that aziridination with imino-λ^3^-bromane CF_3_SO_2_N=BrC_6_H_4_CF_3_ is one of a few methods of synthesis of N-triflylaziridines [15,52,53]. Aziridines can be intermediate products, leading finally to various heterocycles. Thus, 2-alkoxy-3,4-dihydro-2*H*-pyranes react with PhI=NTs with rearrangement and formation of 5-alkoxypyrrolidines [54]. The diversity of possible pathways of the reaction of sulfonamides and their derivatives with alkenes is schematically shown in Scheme 1.

Most of the methods described above require the use of transition metal catalysts. As an alternative, Minakata et al. proposed a new, metal-free procedure for aziridination of alkenes by commercially available sulfonamides as a nitrogen source in the oxidative system (Bu*^t^*OCl + NaI) in up to 95% yield [55]. The active intermediate is Bu*^t^*OI, formed in situ along with other species as was described in an earlier work [56]. In particular, it was found that amidation of *trans*-2-octene proceeded with complete stereoselectivity, leading to the *trans*-substituted aziridine as the sole product; the conclusion was made on the completely stereoselective and stereospecific aziridination, consistent with the reaction mechanism involving a cyclic iodonium intermediate.

**Scheme 2:**
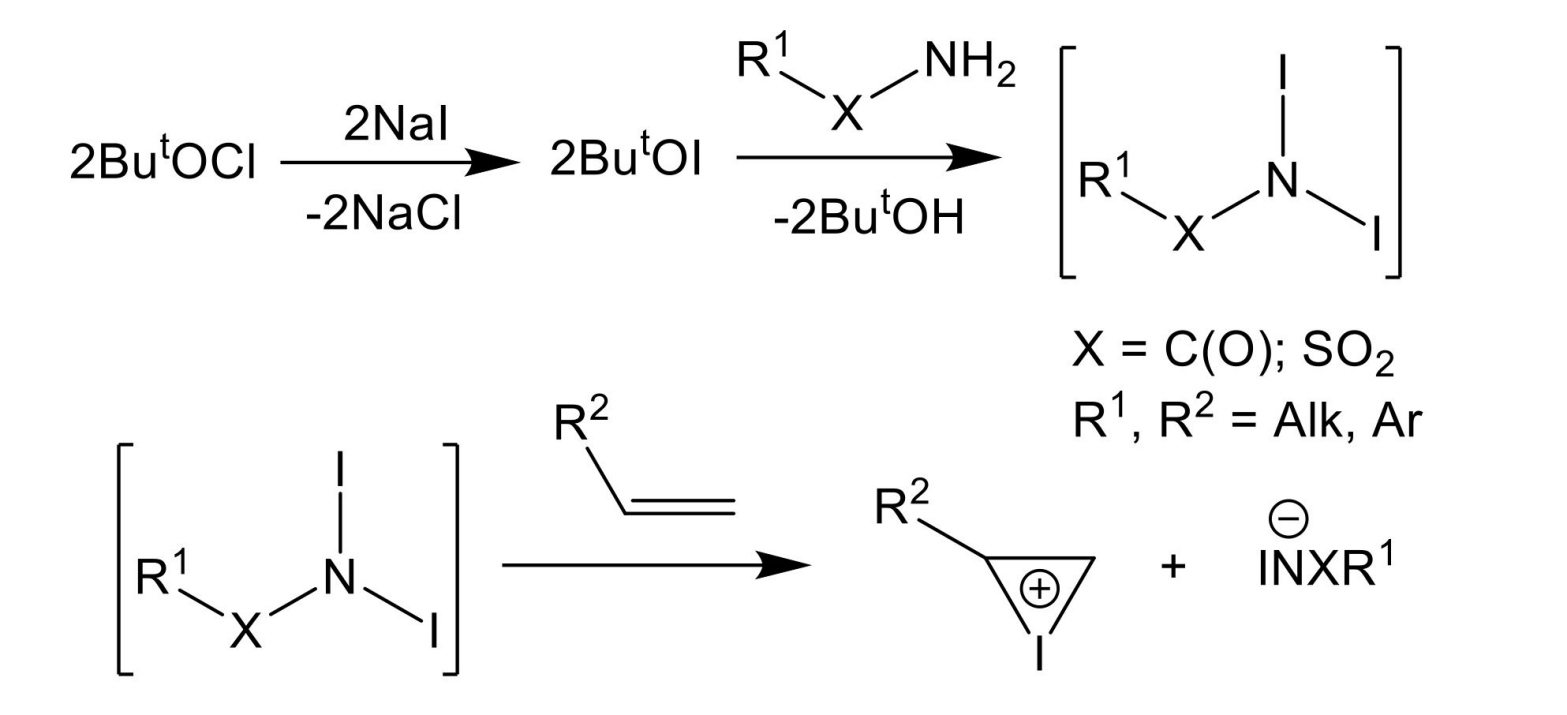
Tentative mechanism of formation of iodonium cations by the reaction of alkenes with *N*,*N*-diiodocarboxamides or -sulfonamides as the key intermediates.

The scale of applicability of Bu*^t^*OI-mediated amidation is not limited by the preparation of aziridines, since the formation of other valuable heterocycles was also reported [3,57,58,59,60,61,62,63,64,65]. Thus, non-activated alkenes when treated with benzamides in presence of Bu*^t^*OI afford oxazolines [55]. The authors proposed a tentative mechanism with participation of the in situ generated molecules of Bu*^t^*OI, which react with the amide, rather than with alkene, to give *N*,*N*-diiodoamides (Scheme 2) [57]. The formation of the latter intermediates was assumed based on the ^1^H-NMR analysis, which showed that the signal of the amide protons gradually disappeared and the signal of the *tert*-butyl group of Bu*^t^*OH appeared within one hour at room temperature. Then, RC(O)NI_2_ react with alkenes to give cyclic iodonium intermediates which capture the amide counter anion. Note the temperature dependence of halogenation of amides: *N*,*N*-dibromotosylamide, generated by the reaction of tosylamide with NBS at 25 °C, was completely transformed to *N*-bromotosylamide upon heating to 40 °C, as proven by ^1^H-NMR; as a result, the opposite amidation regioselectivity was observed [66]. This is an illustration of the necessity of both theoretical and experimental investigation of the Bu*^t^*OI-mediated amidation mechanism.

In continuation of our studies on the sulfamidation of alkenes and dienes under oxidative conditions using Minakata’s reagent, Bu*^t^*OCl + NaI, we recently reported on oxidative sulfamidation of vinyl silanes with triflamide and arenesulfonamides, in which the products of iodochlorination, aziridination and heterocyclization to 1,4-azasilinanes were obtained in high total yield [61]. Diverse reactivity in the reactions of oxidative sulfamidation leading to numerous and unexpected products prompted us to perform a quantum chemical study of the mechanism of oxidative amidation of alkenes in the presence of Bu*^t^*OCl + NaI. As the model substrates, styrene (**1**), trimethyl(vinyl)silane (**2**), dimethyl(divinyl)silane (**3**) and diphenyl(divinyl)silane (**4**) were chosen in the reactions with mesylamide (CH_3_SO_2_NH_2_, **a**), triflamide (CF_3_SO_2_NH_2_ or TfNH_2_, **b**), tosylamide (Tol^p^SO_2_NH_2_ or TsNH_2_, **c**) and *p*-nosylamide (NO_2_C_6_H_4_^p^SO_2_NH_2_ or NsNH_2_, **d**) were used. The performed study does not purport to answer all questions concerning the mechanism of oxidative amidation, but, rather, to shed light on some unresolved experimental observations.

## 2. Computational Details

The geometries of all molecules were optimized at the B3LYP/DGDZVP level of theory using the Gaussian 09 software [67]. For all the atoms, the all-electron, double-ζ valence polarized basis set (DGDZVP) was used. It has been demonstrated that the DGDZVP basis set applied for iodine-containing organic molecules provides a much better description of the activation barriers than other double-ζ basis sets, and it even outperformed the triple-ζ basis sets [68]. Due to its small size, it is well-suited to studying halogen bonding in large systems. Since the Bu*^t^*OI-mediated reactions of oxidative sulfamidation were carried out, as a rule, in acetonitrile, all calculations were also performed for MeCN as a solvent, using the integral equation formalism polarizable continuum model (IEF-PCM) [69]. For each stationary point, frequency calculations were performed to confirm whether these structures were local minima or transition states (TS). All transition state calculations were accompanied by intrinsic reaction coordinate (IRC) calculations to verify that each TS connected the corresponding reactants and products. The Møller–Plesset second order perturbation theory (MP2) was used for energy refinement. Throughout the Results and Discussion section, if not stated otherwise, “energy” (taking into account the zero-point vibrational energy (ZPVE) correction) refers to the MP2/DGDZVP//B3LYP/DGDZVP calculations. For comparison, the long range corrected density functional theory (DFT) functional, which includes empirical dispersion correction wB97XD with the same basis set, was also tested.

## 3. Results and Discussion

### 3.1. Formation of Reactive Iodine-Containing Species

**Scheme 3:**
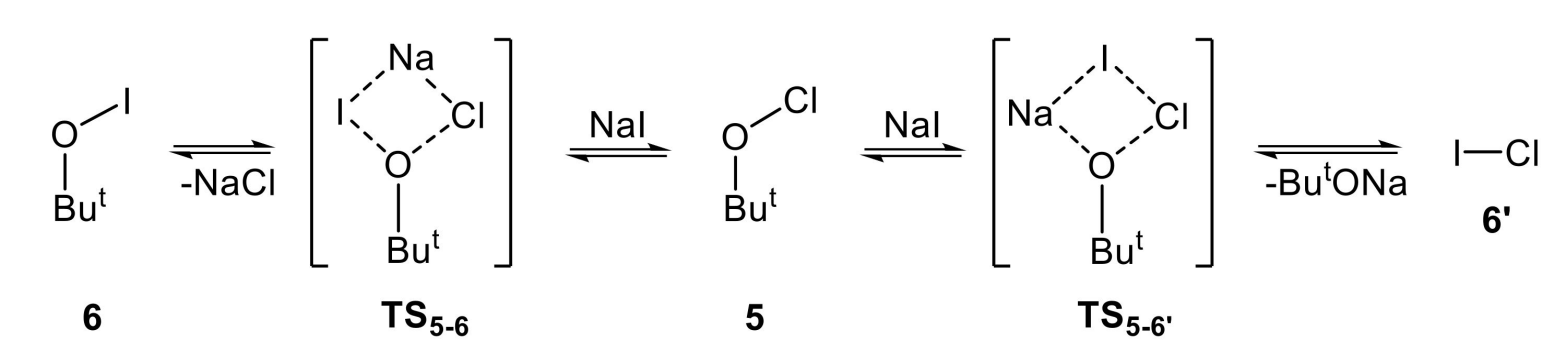
Possible transformations in the system Bu*^t^*OCl + NaI.

Since for a successful reaction between alkenes and sulfonamides the reagents, which must be introduced first into the reaction system, need to be Bu*^t^*OCl (**5**) and NaI, we started with the analysis of the reactions occurring between the two, including the formation of Bu^t^OI (**6**) (Scheme 3).

As follows from Scheme 3, two possible pathways for coordination of NaI to the oxygen atom of Bu*^t^*OCl (**5**) can occur: (i) via the iodine atom, or (ii) via the sodium atom, leading in the formation of Bu*^t^*OI (**5** → **6**) or ICl (**5** → **6’**), respectively. The former reaction proceeds via the transition state **TS_5-6_** (173*i* cm^–1^) lying 28.8 kcal/mol above the reagents (Bu*^t^*OCl + NaI). The energy gain for the formation of Bu*^t^*OI **6** is 10.9 kcal/mol. The **5** ⇆ **6** equilibrium is shifted towards **6** with Δ*G* = −10.5 kcal/mol. We could not locate a transition state for the reaction **5** → **6’**. The forced relaxed stepwise rapprochement of Bu*^t^*OCl and NaI that could lead to the formation of ICl (**6’**) and Bu*^t^*ONa was accompanied by a steady energy increase up to 24.5 kcal/mol. Therefore, the only possible reaction in the system Bu*^t^*OCl + NaI is the formation of Bu*^t^*OI (**6**), while the formation of iodine chloride **6’** apparently occurs in a different way. The results are summarized in Table 1.

### 3.2. The Effect of the Sulfonamide Structure

In view of numerous examples illustrating specific behavior of triflamide, distinct from that of non-fluorinated sulfonamides [58,60,70,71], it was of interest to compare the relative iodinating activity of different *N*-iodinated sulfonamides. We performed such an analysis at the MP2//B3LYP level of theory using as a criterion the ease of the N–I bond rupture in both active intermediates capable of acting as iodinating agents, namely, RSO_2_NHI (**7**) and RSO_2_NI_2_ (**8**). For the following reactions:

(1) $${\mathrm{RSO}}_{2}\mathrm{NHI}\to {\mathrm{RSO}}_{2}{\mathrm{NH}}^{-}{+I}^{+}$$

(2) $${\mathrm{RSO}}_{2}{\mathrm{NI}}_{2}\to {\mathrm{RSO}}_{2}{\mathrm{NI}}^{-}{+I}^{+}$$

where the relative ZPVE-corrected values of Δ*E* decrease (and, hence, the iodinating activity increases) in the following order: RSO_2_ = Ts (16.7) ≥ Ms (16.1) > Ns (12.5) >> Tf (0) kcal/mol for (**7**), and RSO_2_ = Ts (14.8) ≥ Ms (14.3) > Ns (11.0) >> Tf (0) kcal/mol for (**8**). Expectedly, the iodinating activity of RSO_2_NI_2_ (**8**) is slightly (by 1.5–1.9 kcal/mol) higher than that of RSO_2_NHI (**7**).

**Scheme 4:**
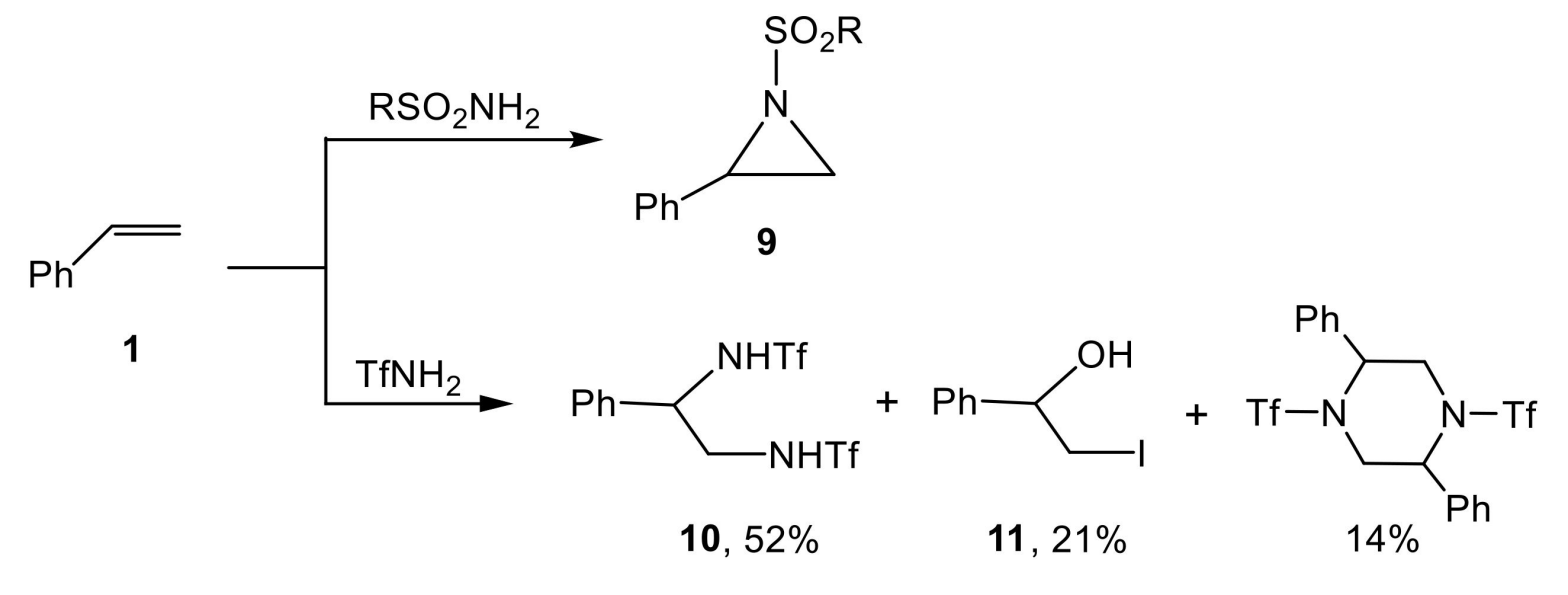
Different courses of the reaction of styrene with triflamide and non-fluorinated sulfonamides. Conditions: (Bu*^t^*OCl + NaI), MeCN, −10 °C.

The performed theoretical analysis also allows shedding light on another issue: the dependence of the product composition on the structure of the sulfonamide. Thus, while the reactions of non-fluorinated sulfonamides with various alkenes, including styrene **1**, in the system Bu*^t^*OCl + NaI gives rise only to aziridines **9** [55], from the reaction of styrene with triflamide the main product of bis-amidation **10** and a substantial amount (21%) of iodoalcohol PhCH(OH)CH_2_I **11** were isolated [72] (Scheme 4).

To rationalize this unexpected result, we performed calculations of the two following reactions, focusing not on the absolute values of ΔΔ*E* between the two reactions, which itself does not have much sense, but rather on its variation with substituent R:

(3) $${\mathrm{RSO}}_{2}{\mathrm{NH}}_{2}{+\mathrm{Bu}}^{t}\mathrm{OI}\to {\mathrm{RSO}}_{2}{\mathrm{NHI}+\mathrm{Bu}}^{t}\mathrm{OH}$$

(4) $${1+\mathrm{Bu}}^{t}{\mathrm{OI}+H}_{2}O\to {11+\mathrm{Bu}}^{t}\mathrm{OH}$$

The energetic prevalence of the latter process for the reaction with triflamide is much higher than for non-fluorinated sulfonamides [CF_3_ (4.3) >> CH_3_ (1.8) > *p*-NO_2_C_6_H_4_ (0.7) ≥ *p*-CH_3_C_6_H_4_ (0) kcal/mol] suggesting that the formation of iodoalcohol is relatively much more favorable with triflamide than with other sulfonamides. This is consistent with its formation in our work [72], and with the literature data on its absence in the reaction with non-fluorinated sulfonamides [55].

**Scheme 5:**
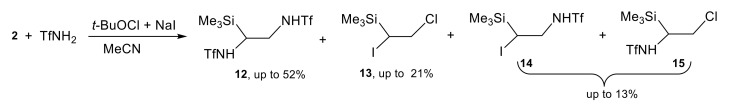
Different regioselectivity of halotriflamidation of trimethyl(vinyl)silane **2**.

No less interesting is a question about different regioselectivity of halogenation of styrene **1** and trimethyl(vinyl)silane **2**. While the former gives only the β-iodinated product **11** (Scheme 4), compound **2** affords, apart from the products of bis-amidation **12** and dihalogenation **13**, both the α- and β-halogenated products **14**, **15** in comparable (although small) yields [61] (Scheme 5).

**Figure 1:**
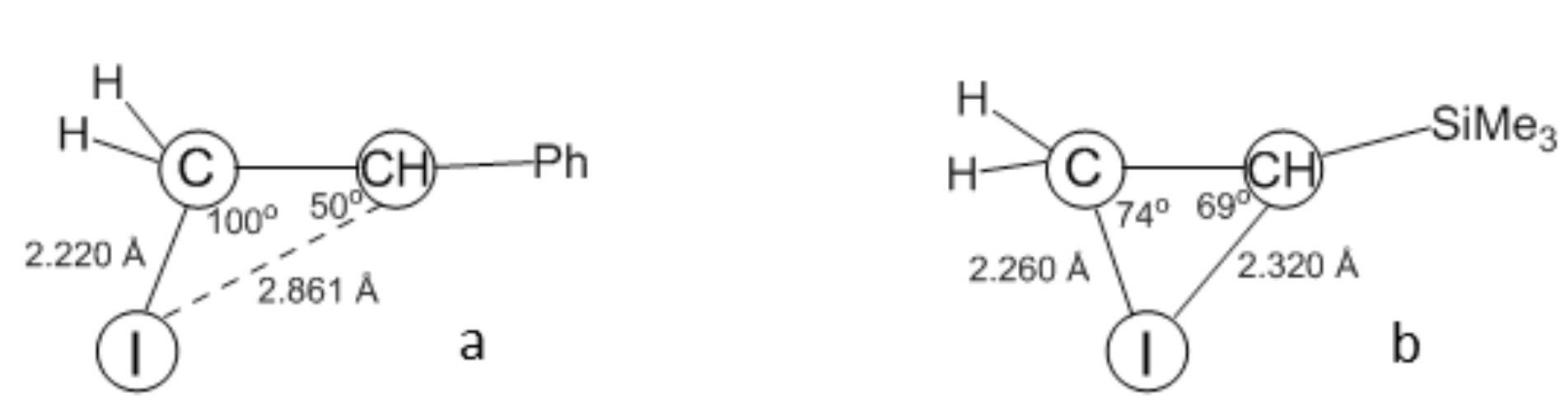
Linear (a) and cyclic (b) iodonium cations from electrophilic iodination of styrene **1** and trimethyl(vinyl)silane **2**.

The calculations give an explicit answer to the question about this apparent discrepancy. The reason is the different structure of the corresponding iodonium cations: almost linear cation in the case of styrene and practically cyclic one in the case of silane **2** as follows from the comparison of the bond lengths and bond angles in Figure 1.

The linear (left) structure in Figure 1 is stabilized as the benzylic type cation, which can add a nucleophile only to the α-carbon affording the β-iodinated product, e.g., **11**. The cyclic (right) structure is due to the enhanced electron density on the vinyl group of silane **2** owing to the electronodonor effect of the Me_3_Si group, and can add the amide anion to either of the two carbon atoms resulting in both α- and β-halogenated products, as was experimentally proven (Scheme 5).

**Scheme 6:**
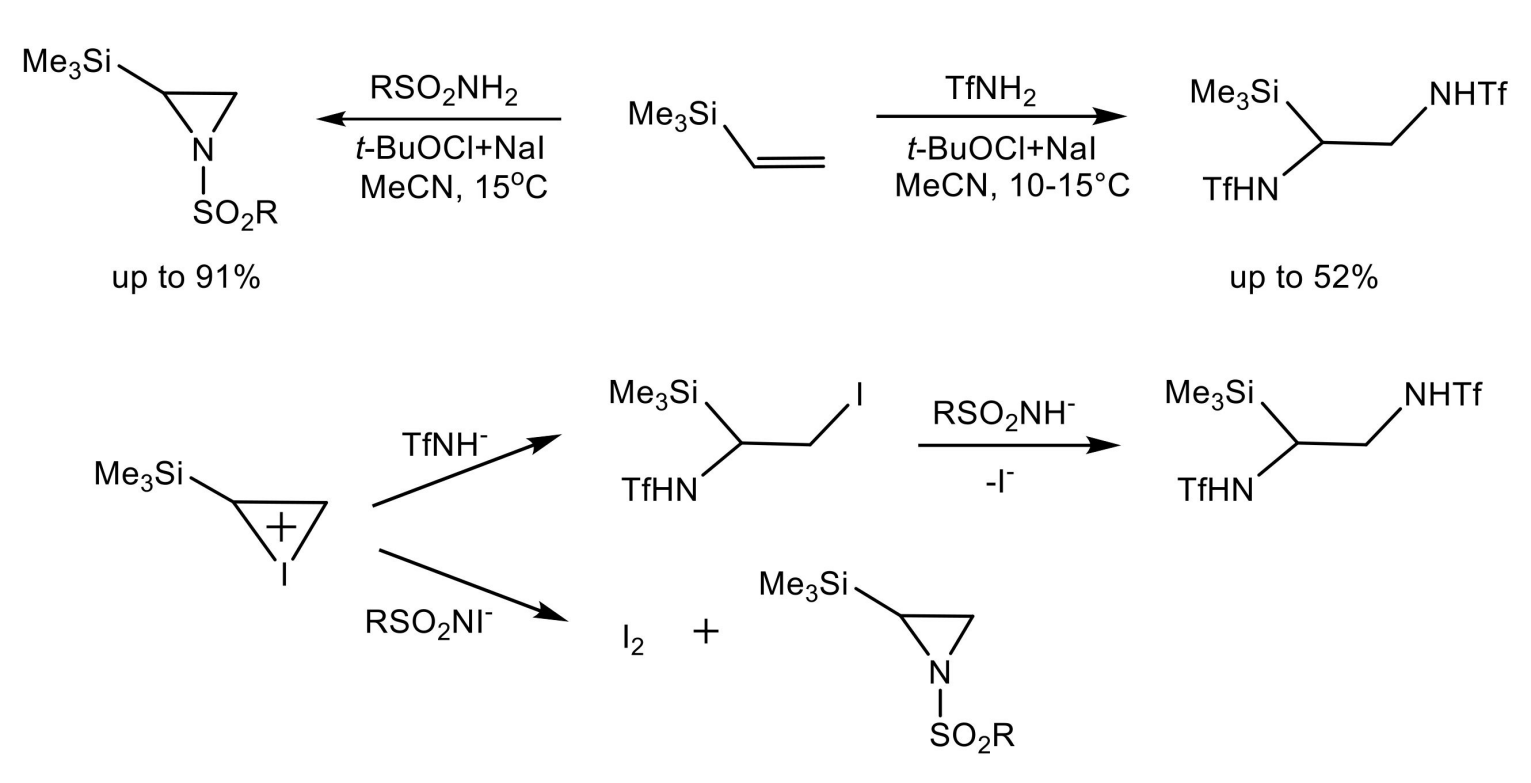
Different courses of the reaction of trimethylvinylsilane with triflamide and non-fluorinated sulfonamides.

The most challenging difference between the reactivities of non-fluorinated sulfonamides and triflamide is the formation of aziridines from styrene **1** or silane **2** as the only products for the former and the main products of bis-triflamidation of **1**, **2**, and some other alkenes for triflamide [61,72,73], as shown in Scheme 6 on the example of silane **2**.

**Scheme 7:**
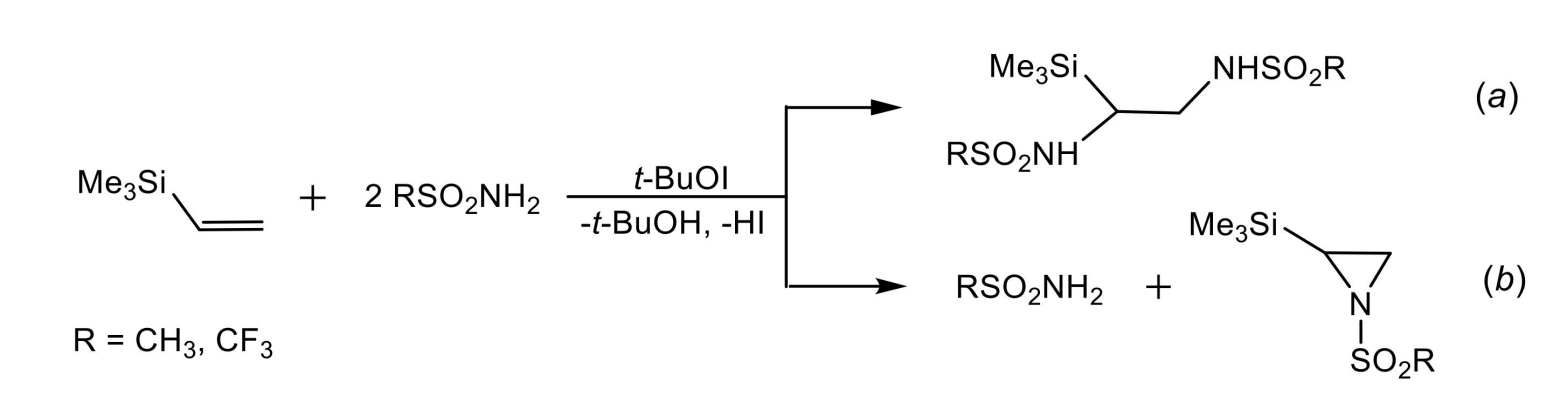
Bis-sulfamidation versus aziridination of trimethyl(vinyl)silane.

Similar behavior of substantially different substrates in Scheme 4; Scheme 6 proves the generality of the conclusion about different reactivity of triflamide and its nonfluorinated analogues and requires reasonable explanation. For this, we have calculated the reactions of bis-sulfamidation and aziridination for mesylamide and triflamide on the example of silane **2** and compared the thermodynamics of routes (*a*) and (*b*) in Scheme 7. 

Due to the same composition of the products in Scheme 7, such an approach allows, without loss of generality, to avoid the analysis of numerous possible transition states and intermediates by comparing the free energies for the two pathways. Because of the high ring strain of the aziridine ring, route (*a*) is energetically 26.4 kcal/mol preferable over route (*b*) for R = CH_3_, and even more preferable (by 29.5 kcal/mol) for R = CF_3_. The entropy factor acts in the opposite direction and decreases the value of Δ*G* with respect to Δ*E* from −26.4 to −9.8 kcal/mol for R = CH_3_ and from −29.5 to −14.0 kcal/mol for R = CF_3_. Therefore, the entropy factor also favors route (*a*), the value of ΔΔ*G* being 4.2 kcal/mol. Such a notable free energy difference explains the experimentally observed formation of linear adducts in the reactions with triflamide [61,72] and of aziridines with non-fluorinated sulfonamides [55].

### 3.3. Interactions between Bu^t^OCl(I), Alkene and Sulfonamide

The next step was to investigate possible interactions of Bu^t^OCl(I) and the model alkenes **3** and **4** with various sulfonamides. The approaching of Bu*^t^*OCl (**5**) to dimethyl(divinyl)silane (**3**) and diphenyl(divinyl)silane (**4**) is slightly exothermic by 3.0 and 3.8 kcal/mol, respectively, resulting in the formation of π-complexes with one of the vinyl groups; the formation of similar complexes with Bu*^t^*OI (**6**) is twice as exothermic as that (5.9 and 7.4 kcal/mol, respectively). However, the Gibbs free energy slightly increases due to the loss of entropy, see Table 2.

**Scheme 8:**
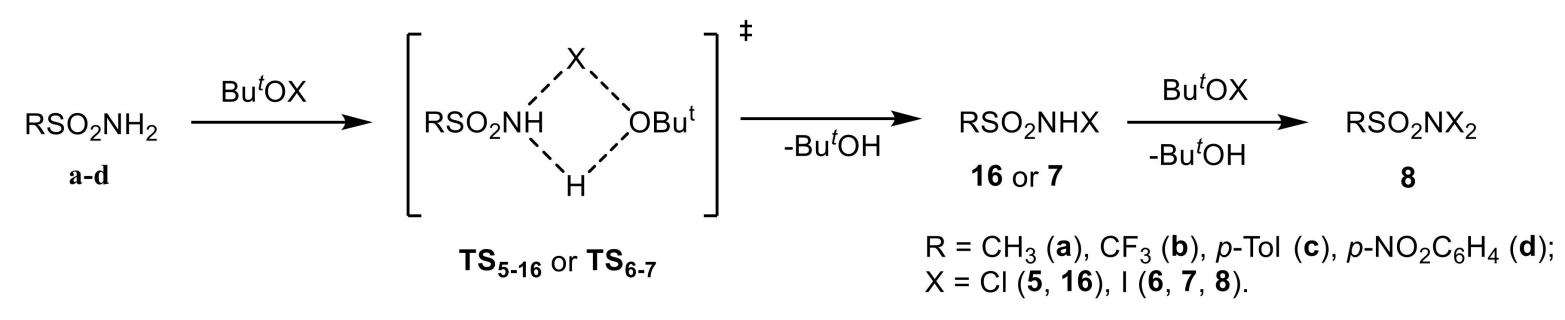
Halogenation of sulfonamides with Bu^t^OI(Cl).

The reaction of halogenating agents Bu*^t^*OCl(I) with the model sulfonamides **a–d** proceeds as shown in Scheme 8, via the transition state **TS_5-16_** or **TS_6-7_** (Figure S1). The transition states **TS_5-16_** corresponding to the formation of N-chlorosulfonamides (**16**) lie very high in energy, from 59.5 (**d**) to 64.0 kcal/mol (**a**), suggesting a highly improbable reaction. Still, the formation of products **16a–d** is slightly exothermic, with the energy gain varying from 1.8 (**b**) to 6.6 kcal/mol (**c** or **d**, see Table S1).

In contrast, iodination of sulfonamides **a–d** with Bu*^t^*OI (**6**) occurring via the transition state **TS_6-7_** is connected with overcoming a much lower activation barrier from 28.6 (**b**) to 32.0 kcal/mol (**a**) with respect to the nonreacting system (**5** + sulfonamide **a–d**), which is about twice as low as that for chlorination through the transition state **TS_5-16_** (Scheme 8, Table S2). Hence, the iodination of sulfonamides with Bu*^t^*OI proceeds much easier than their chlorination with Bu*^t^*OCl, in spite of lower N–I energy bond with respect to N–Cl bond (by ~5 kcal/mol). This means that the ease of halogenation is determined by the ease of the O–Hlg bond rupture, rather than the N–Hlg bond formation. *N*-Iodosubstituted non-fluorinated sulfonamides **7 a**,**c**,**d** lie 0.2–2.4 kcal/mol lower, but, for N-iodotriflamide **7b**, it lies 2.2 kcal/mol higher with respect to the unreacted system (sulfonamide + Bu^t^OI). Further iodination affording *N*,*N*-diiodosulfonamides **8** through **TS_7-8_** proceeds by ~7 kcal/mol easier than the first one. The equilibrium in the system (sulfonamide + 2Bu*^t^*OI ⇆ **8** +2Bu^t^OH) is slightly shifted to the products, the Gibbs free energy Δ*G* varying from 0.8 (**b**) to 3.1 kcal/mol (**c**, **d**), and the ZPVE-corrected total energies of the products Δ*E* being from 0.7 (**b**) to 4.4 kcal/mol (**c**) below the reagents (Table S2). Taking into account the fact that Bu*^t^*OI is formed in the reaction of Bu*^t^*OCl with NaI, which is exothermic by 10.9 kcal/mol (Table 1), the relative energy of *N*,*N*-diiodosulfonamides **8** must be 2 × 10.9 = 21.8 kcal/mol below the energy of the unreacted system (2Bu^t^OCl + 2NaI + sulfonamide).

### 3.4. Analysis of Reactivity of Sulfonamides, Mono- and Diiodosulfonamides

Some geometric parameters of sulfonamides **a–d**, *N*-iodosulfonamides **7**, and *N*,*N*-diiodosulfonamides **8** as well as some electronic effects in their molecules are presented in Table 3.

As follows from Table 3, with the increase of the iodine atoms number (*n*) from 0 to 2, the S–N bond in sulfonamides is elongated (weakened). Simultaneously, the pyramidality of the nitrogen atom decreases indicating the increase of the *p*-character of the nitrogen lone pair from sp^2.9^ to sp^2.5^ (R = Me, Ar) or from sp^2.5^ to sp^2.2^ (R = CF_3_). The HOMO–LUMO energy gap narrows from the maximum value of 18.4 eV (R = CF_3_, *n* = 0) to the minimum of 9.4 eV (R = *p*-Tol, *n* =2), while the LUMO goes down and in *N*,*N*-diiodosulfonamides (*n* = 2), it lies below zero (~ –0.4 eV), being localized on the antibonding iodine atom lone pair. Note that the MO localized on the π-bond of the vinyl group in **3** corresponds to HOMO and lies at –9.97 eV, whereas in **4** it corresponds to HOMO-4 and lies at –10.32 eV. With the increase of the iodine atoms, the electron density on the nitrogen atom decreases, whereas its variations on the iodine atom are negligible.

**Scheme 9:**
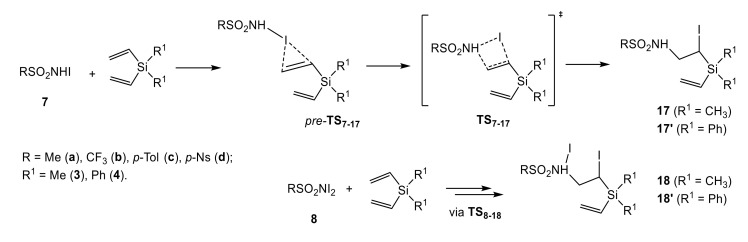
Formation of the products of iodosulfamidation by the reaction of *N*-iodo- (**7**) and *N*,*N*-diiodosulfonamides (**8**) with dimethyldivinyl- (**3**) and diphenyldivinylsilane (**4**).

*N*-Iodosulfonamides **7** are capable of forming π-complexes with the vinyl group of substrates **3** and **4** with lowering the energy of the system by ~7 kcal/mol as compared to unreacted system [**7** + **3** (or **4**)] (Table S3). These π-complexes are pre-reaction complexes on the potential energy surface of the reaction of iodosulfamidation (Scheme 9). The transition states of the reaction of iodosulfamidation of **3** (**4)**, **TS_7-17_**, lie 23 kcal/mol (20 kcal/mol) above the unreacted system [**7** + **3** (or **4**)]. The products of iodosulfamidation of **3** or **4** (**17** or **17′**) lie ~45 kcal/mol below the unreacted system. *N*,*N*-Diiodosulfonamides **8** demonstrate similar reactivity; however, the reaction with **3** or **4** proceeds much easier because the barrier heights (**TS_8-18_**) are lower than those of **TS_7-17_**, with the activation energies from 4.7 (**c**) to 9.5 kcal/mol (**b**) in the case of **TS_8-18_** and from 1.6 (**d**) to 9.0 kcal/mol (**b**) in the case of **TS_8-18’_** (Table S4). The adducts of *N*,*N*-diiodosulfonamides with **3** or **4**—*N*-(2-(dimethyl(vinyl)silyl)-2-iodoethyl)-*N*-iodosulfonamides **18** or *N*-(2-(diphenyl(vinyl)silyl)-2-iodoethyl)-*N*-iodosulfonamides **18’** lie ~46 kcal/mol below the nonreacting system (**8** + **3** or **4**).

**Scheme 10:**
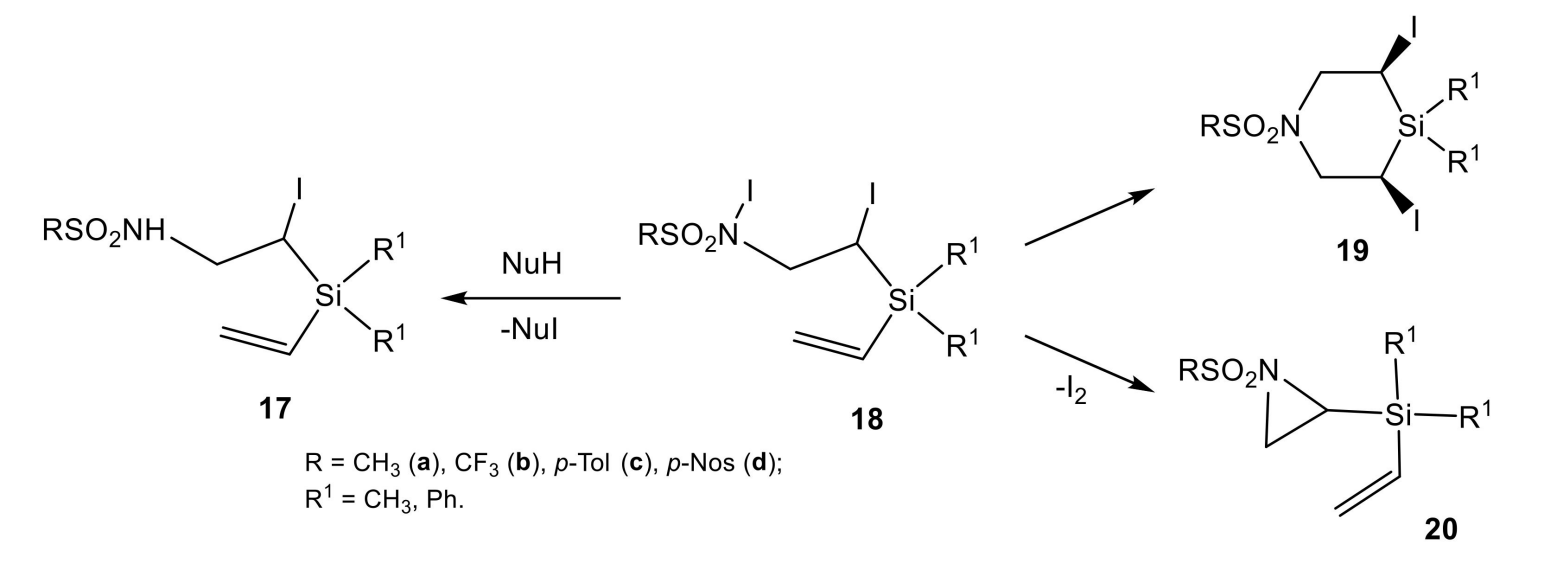
Possible transformations of N-(2-((vinyl)silyl)-2-iodoethyl)-*N*-iodosulfonamides **18**.

Adducts **18** or **18’** are key intermediates for further development of the reaction, since they can (i) act as strong iodinating agents for sulfonamides, monoiodosulfonamides and other species in the reaction mixture; (ii) add intramolecularly to the second C=C bond due to the presence of the N-I moiety forming 1,4-azasilinanes; (iii) eliminate molecular iodine to give *N*-sulfonylaziridines (Scheme 10).

The iodinating properties of **18** are due to a weak N–I bond (<40 kcal/mol) and are manifested, e.g., in the exchange reactions with sulfonamides or *N*-iodosulfonamides, or with Bu*^t^*OH to recover Bu*^t^*OI. The **18** → **17** reaction proceeds through **TS_18-17_** with a very low activation barrier of only circa 4 kcal/mol in the forward direction in the case of **18b**. A similar reaction with **18a** has an even smaller barrier, of circa 3 kcal/mol.

**Figure 2:**
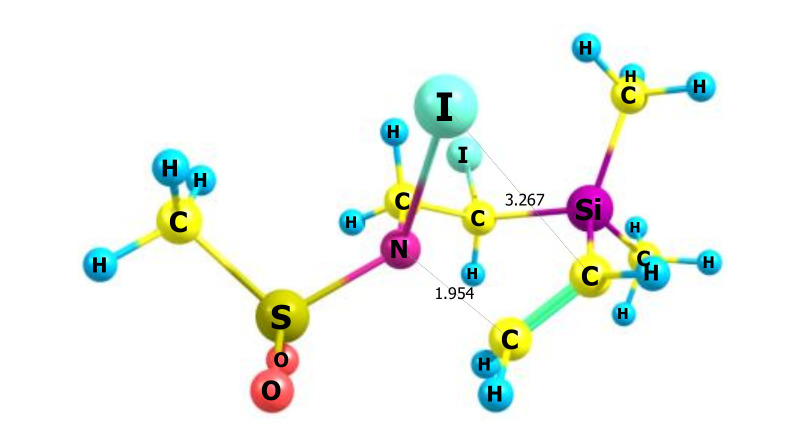
The structure of the transition state **TS_18-19_**, R = R^1^ = CH_3_.

On the other hand, the N–I iodine atom in **18** can form intramolecular π-complex with the intact vinyl group with subsequent cyclization to 1,4-azasilinanes **19**. The corresponding transition state **TS_18-19_** was also found, Figure 2. The **18** → **19** reaction has activation barrier of ~30 kcal/mol in the forward direction; its driving force is the formation of a stable product of cyclization—(3*R*,5*S*)-3,5-diiodo-1-organylsulfonyl-1,4-azasilinanes **19** (twist conformer), which lies 33–38 kcal/mol below adducts **18** depending on substituents R and R^1^ (Table S5). We could not locate **TS_18-19_** for R = CF_3_ and R^1^ = CH_3_; instead, cyclization occurred with synchronous elimination of CF_3_SO_2_. For R^1^ = Ph, the corresponding **TS_18-19_** was found to lie 50 kcal/mol above **18′b** making this transformation impossible. Note that product **19** is formed stereospecifically and, initially, in the twist conformation of the ring, which is unfavorable as compared to “chair” by 5–7 kcal/mol.

**Figure 3:**
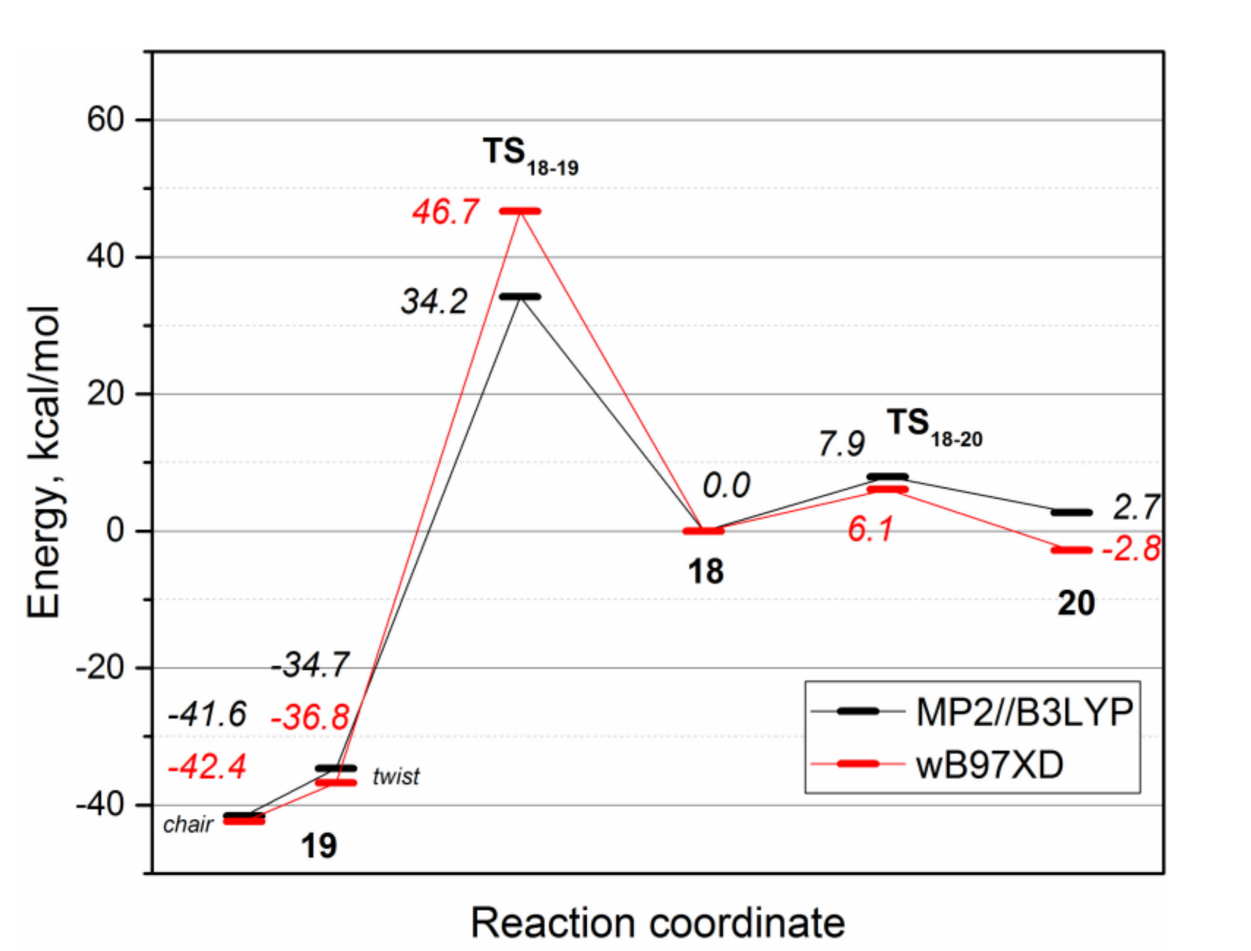
Energy diagram for conversion of adduct **18** to 1,4-azasilinane **19** and aziridine **20**. R = R^1^ = CH_3_ (refer to Scheme 10 for details).

Finally, adducts **18** can eliminate molecular iodine to afford aziridines **20** (Scheme 9, Table S5). The reaction is preceded by elimination of the iodine atom from nitrogen in **18** via **TS_18-20_**. The **18** → **20** activation barriers are only 6–10 kcal/mol in the forward direction. Again, **TS_18-20_** for R =CF_3_ could not be located for both R^1^ = CH_3_ and Ph. Aziridines **20** lie ~3.8 kcal/mol (in average) higher than **18** at the MP2//B3LYP level of theory, whereas wB97XD, vice versa, predicts it to be 2.8 kcal/mol more favorable (for R = R^1^ = CH_3_). The energy diagram for transformation of adducts **18** for R = R^1^ = CH_3_ is shown in Figure 3.

## 4. Conclusions

The analysis of the sulfamidation of different substrates with various sulfonamides in the oxidative system (Bu*^t^*OCl + NaI) was performed at the MP2/DGDZVP//B3LYP/DGDZVP level of theory with the solvent effect taken into account by the IEF-PCM method for MeCN as a solvent. The only energetically favorable process in the aforesaid system is the formation of Bu*^t^*OI acting as an active intermediate generating the mono- and diiodinated sulfonamides RSO_2_NHI and RSO_2_NI_2_ from the reagents. Judged from the ease of the N–I bond rupture with generation of electrophilic I^+^ in the two species, their iodinating activity increases in the order RSO_2_: Ts ≥ Ms > Ns >> Tf being 1.5–1.9 kcal/mol higher for RSO_2_NI_2_ than for RSO_2_NHI. The intriguing difference between the reactivity of triflamide forming mainly the products of bis-triflamidation and the non-fluorinated sulfonamides leading to aziridines is found to be due to different thermodynamics of the two routes. The energetic preference of addition over cyclization caused by a high strain of the aziridine ring is by 3.1 kcal/mol larger for triflamide than for mesylamide. Smaller entropy loss for triflamide increases this difference to ΔΔ*G* of 4.2 kcal/mol, thus explaining the experimental observations. Different regioselectivity of oxidative addition to styrene and trimethyl(vinyl)silane is explained by a different structure of the intermediate iodonium cations: a linear one formed from styrene and leading to the β-iodinated product, and a cyclic one formed from vinylsilane and opened by nucleophilic attack of RSO_2_NH^–^ on the sterically less hindered β-carbon atom. The mechanism of the reaction of non-fluorinated sulfonamides RSO_2_NH_2_ with divinylsilanes **3** and **4** in the oxidative system (Bu*^t^*OCl + NaI) has been studied in detail. The reaction results in the formation of *N*-sulfonylaziridines and 1,4-azasilinanes as the kinetic and thermodynamic products, respectively. The adducts of *N*,*N*-diiodosulfonamides to one of the vinyl groups of the silane are key intermediates of the reaction.

## Figures and Tables

**Table 1:** Relative energies (kcal/mol) of molecules **6** and **6’** formed in the system Bu*^t^*OCl + NaI.

Parameter	5	TS_5-6_	6	6’
MP2/DGDZVP//B3LYP/DGDZVP				
Δ*E+ZPVE*	0.0	28.8	−10.9	24.5
Δ*G*	0.0	38.8	−10.5	23.6
wB97XD/DGDZVP				
Δ*E+ZPVE*	0.0	25.4	−13.0	20.0
Δ*G*	0.0	34.3	−12.8	19.4

**Table 2:** Relative energies (kcal/mol) of π-complexes of divinylsilanes **3**, **4** with *t*-BuOCl **5** and *t*-BuOI **6**.

Parameter	5 + 3 or 4	5···3	5···4	6 + 3 or 4	6···3	6···4
MP2/DGDZVP//B3LYP/DGDZVP						
Δ*E+ZPVE*	0.0	−3.0	−3.8	−10.9	−16.8	−18.3
Δ*G*	0.0	6.9	5.0	−10.5	−6.0	−7.7
wB97XD/DGDZVP						
Δ*E+ZPVE*	0.0	–	–	−13.0	−19.2	−21.2
Δ*G*	0.0	–	–	−12.8	−8.6	−8.3

**Table 3:** Geometric and electronic parameters of sulfonamides, mono- and diiodosulfonamides RSO_2_X, X = NH_2–n_I_n_ (MP2/DGDZVP//B3LYP/D GDZVP). HOMO (highest occupied molecular orbital), LUMO (lowest unoccupied molecular orbital), CM5 (charge model 5), NBO (natural bond order), LP (lone pair).

Parameter	R = CH_3_			R = CF_3_			R = *p*-Tol			R = *p*-NO_2_C_6_H_4_		
	X= NH_2_	NHI	NI_2_	NH_2_	NHI	NI_2_	NH_2_	NHI	NI_2_	NH_2_	NHI	NI_2_
S–N, Å	1.678	1.707	1.745	1.646	1.659	1.679	1.683	1.717	1.761	1.673	1.702	1.740
N–I, Å	–	2.115	2.136	–	2.103	2.121	–	2.116	2.139	–	2.111	2.134
∠SNI(H)I(H)	126.5	126.6	137.0	135.1	141.0	155.3	124.9	123.7	135.5	127.7	127.4	138.6
Σ_N_	335.5	337.3	344.9	343.3	348.6	355.2	333.8	334.6	343.7	336.6	338.1	346.0
HOMO, eV	−12.720	−10.480	−10.242	−13.453	−10.685	−10.382	−9.734	−9.835	−9.881	−10.375	−10.349	−10.289
LUMO, eV	5.141	0.644	−0.475	4.946	0.317	−0.735	2.161	0.694	−0.435	0.197	0.116	−0.545
LUMO–HOMO gap, eV	17.861	11.124	9.767	18.398	11.001	9.647	11.895	10.529	9.449	10.571	10.466	9.744
CM5 charge on N	−0.600	−0.503	−0.412	−0.574	−0.501	−0.428	−0.601	−0.502	−0.407	−0.593	−0.501	−0.412
CM5 charge on I	–	0.223	0.223	–	0.279	0.272	–	0.208	0.212	–	0.230	0.232
Electrophilicity (I)	–	1.866	1.600	–	2.168	1.820	–	1.819	1.538	–	1.986	1.568
NBO (LP_N_ occupancy)	1.900	1.909	1.918	1.866	1.867	1.867	1.897	1.908	1.918	1.888	1.898	1.907

## Supplementary Materials

The following are available online, Figure S1 (structure of TS_5-16_, TS_6-7_ for R = CH_3_.), Tables S1–S5 (relative total and free energies of intermediates of transformations **5**→**16**, **6**→**8**, **7**→**17**(**17’**), **8**→**18**(**18’**), **18**→**19**(**20**) and **18****′**→**19****′**(**20****′**).
