# Supplementary material for: Trifluoromethanesulfonamide vs. Non-Fluorinated Sulfonamides in Oxidative Sulfamidation of the C=C Bond: An In Silico Study
Source: Molecules. 2020 Oct 22;25(21):4877. doi: 10.3390/molecules25214877 (PMC7660106; doi:10.3390/molecules25214877)
Supplement: Supplementary file 1 [file molecules-25-04877-s001.pdf]

Trifluoromethanesulfonamide vs. Non-Fluorinated Sulfonamides in Oxidative Sulfamidation of the C=C Bond: an *In Silico* Study

Anton V. Kuzmin, Mikhail Yu. Moskalik, Bagrat A. Shainyan*

* Correspondence: A.E. Favorsky Irkutsk Institute of Chemistry, Siberian Division of Russian Academy of Sciences, 664033 Irkutsk, Russia. E-mail: bagrat@irioch.irk.ru

Received: date; Accepted: date; Published: date

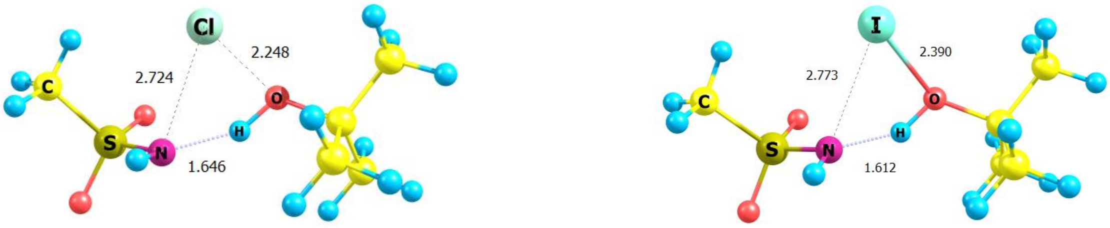

271*i* cm^{-1}
a

245*i* cm^{-1}
b

Figure 1. Structure of transition states **TS$_{5\text{-}16}$** (**a**) and **TS$_{6\text{-}7}$** (**b**) for R = CH_3.

Table 1. Relative energies Δ*E* (ZPVE corrected) and free energies Δ*G* (in kcal/mol) of the intermediates of **5** → **16** transformation.

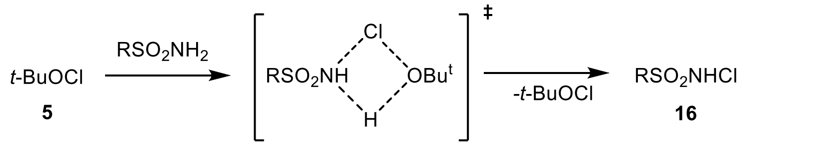

TS$_{5\text{-}16}$ R = CH_3 (**a**), CF_3 (**b**), *p*-Tol (**c**), *p*-Ns (**d**).

Parameter	5 + [a-d]	TS$_{5\text{-}16}$				16			
		a	b	c	d	a	b	c	d
		MP2/B3LYP							
Δ*E*+ZPVE	0.0	64.0	60.4	61.1	59.5	–6.0	–1.8	–6.6	–6.6
Δ*G*	0.0	74.4	71.4	72.7	69.6	–6.4	–1.9	–6.5	–7.6
		wB97XD							
Δ*E*+ZPVE	0.0	59.9	59.7	58.2	58.0	–5.9	–1.8	–5.9	–6.0
Δ*G*	0.0	71.3	71.1	70.4	69.2	–6.2	–2.0	–5.8	–6.6

Table 2. Relative energies ΔE (ZPVE corrected) and free energies ΔG (in kcal/mol) of the intermediates of **6** → **8** transformation.

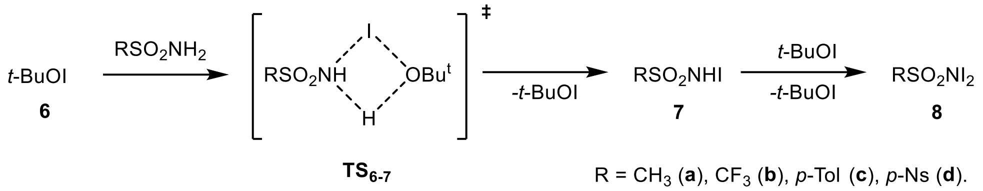

Parameter	6 + [a-d]	TS$_{6-7}$				7				TS$_{7-8}$				8			
		a	b	c	d	a	b	c	d	a	b	c	d	a	b	c	d
								MP2/B3LYP									
ΔE+ZPVE	–10.9	28.8	26.1	26.8	26.3	–11.1	–8.7	–13.3	–12.2	21.2	17.7	20.4	18.5	–13.8	–11.6	–15.2	–15.0
ΔG	–10.5	40.3	38.5	39.2	38.5	–11.1	–8.2	–13.5	–12.8	33.5	29.8	34.1	31.1	–13.0	–11.3	–13.5	–13.6
								wB97XD									
ΔE+ZPVE	–13.0	23.4	–	–	–	–13.8	–12.2	–15.4	–14.5	15.8	–	–	–	–13.4	–12.1	–14.1	–13.7
ΔG	–12.8	35.5	–	–	–	–1.1	0.9	–1.6	–2.2	28.3	–	–	–	–12.7	–11.7	–13.4	–12.3

Table 3. Relative energies ΔE (ZPVE corrected) and free energies ΔG (kcal/mol) of the intermediates of **7** → **17(17′)** transformation.

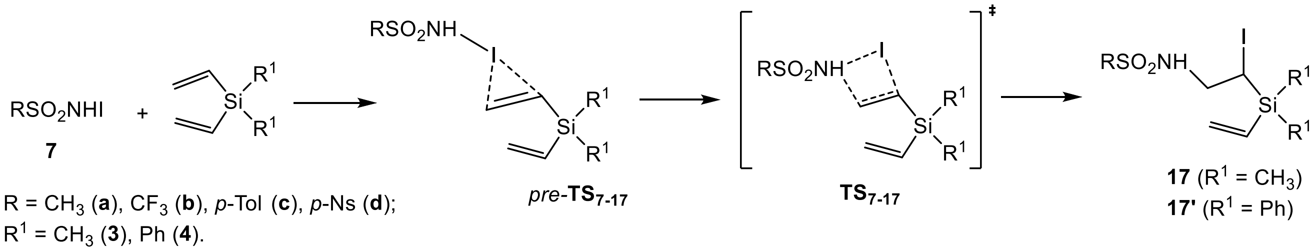

Parameter	7 + 3	*pre*-TS$_{7-17}$				TS$_{7-17}$				17			
						**R^1 = CH$_3$**							
		a	b	c	d	a	b	c	d	a	b	c	d
						MP2/B3LYP							
ΔE+ZPVE	0.0	–5.8	–8.6	–5.7	–6.3	23.2	23.8	21.9	22.8	–44.4	–48.2	–44.6	–45.1
ΔG	0.0	3.8	2.2	5.8	4.2	35.0	35.4	34.3	34.2	–31.3	–35.5	–30.6	–32.0
						wB97XD							
ΔE+ZPVE	0.0	–5.6	–7.6	–7.4	–5.8	34.2	35.8	33.7	34.0	–44.7	–47.9	–45.3	–45.6
ΔG	0.0	4.8	4.6	4.9	5.2	46.7	48.4	44.7	46.8	–31.7	–34.7	–32.2	–32.1
						R^1 = Ph							
Parameter	7 + 4	*pre*-TS$_{7-17'}$				TS$_{7-17'}$				17′			
		a	b	c	d	a	b	c	d	a	b	c	d
						MP2/B3LYP							
ΔE+ZPVE	0.0	–6.7	–9.3	–6.7	–7.1	21.4	20.9	18.9	18.4	–43.4	–47.3	–43.5	–44.5
ΔG	0.0	3.9	2.1	4.9	3.6	33.0	33.2	33.5	34.2	–29.7	–32.2	–28.6	–30.5
						wB97XD							
ΔE+ZPVE	0.0	–6.2	–	–	–	31.5	–	–	–	–43.0	–	–	–
ΔG	0.0	5.1	–	–	–	45.7	–	–	–	–28.6	–	–	–

Table S4. Relative energies Δ*E* (ZPVE corrected) and free energies Δ*G* (kcal/mol) of the intermediates of **8** → **18(18′)** transformation.

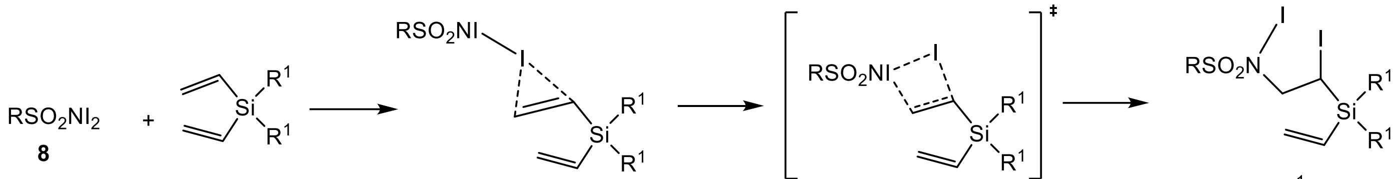

R = CH_3 (**a**), CF_3 (**b**), *p*-Tol (**c**), *p*-Ns (**d**); R^1 = CH_3 (**3**), Ph (**4**).

pre-**TS$_{8\text{-}18}$**

TS$_{8\text{-}18}$

18 (R^1 = CH_3)
18' (R^1 = Ph)

						**R^1 = CH_3**						
**Parameter**	**8 + 3**	***pre*-TS$_{8\text{-}18}$**				**TS$_{8\text{-}18}$**				**18**		
		**a**	**b**	**c**	**d**	**a**	**b**	**c**	**d**	**a**	**b**	**c**	**d**
						MP2/B3LYP						
ΔE+ZPVE	0.0	–8.3	–12.6	–8.2	–9.0	6.0	9.5	4.7	4.8	–44.6	–47.9	–44.8	–45.4
ΔG	0.0	2.3	–1.7	3.2	2.5	18.0	21.9	17.5	17.3	–32.1	–34.9	–31.8	–32.3
						wB97XD						
ΔE+ZPVE	0.0	–7.0	–9.4	–6.9	–7.9	25.2	28.1	25.7	25.9	–45.5	–47.8	–45.8	–46.5
ΔG	0.0	4.0	2.2	4.5	5.0	37.6	41.5	38.9	38.7	–32.9	–34.3	–33.1	–34.2
						R^1 = Ph						
Parameter	**8 + 4**	***pre*-TS$_{8\text{–}18'}$**				**TS$_{8\text{–}18'}$**				**18′**		
		**a**	**b**	**c**	**d**	**a**	**b**	**c**	**d**	**a**	**b**	**c**	**d**
						MP2/B3LYP						
ΔE+ZPVE	0.0	–9.0	–13.5	–9.4	–10.1	5.6	9.0	4.3	1.6	–44.4	–47.5	–44.8	–45.2
ΔG	0.0	2.3	–1.5	1.1	1.1	18.2	21.7	17.3	14.1	–30.8	–33.8	–30.9	–32.0
						wB97XD						
ΔE+ZPVE	0.0	–8.2	–9.7	–8.5	–9.0	24.1	26.4	24.3	24.3	–44.0	–46.2	–44.6	–45.0
ΔG	0.0	4.3	4.1	4.8	3.8	37.6	42.0	38.5	38.0	–29.8	–31.0	–31.4	–30.6

Table S5. Relative energies *ΔE* (ZPVE corrected) and free energies *ΔG* (kcal/mol) of the intermediates of **18** → **19(20)** and **18′** → **19′(20′)** transformation.

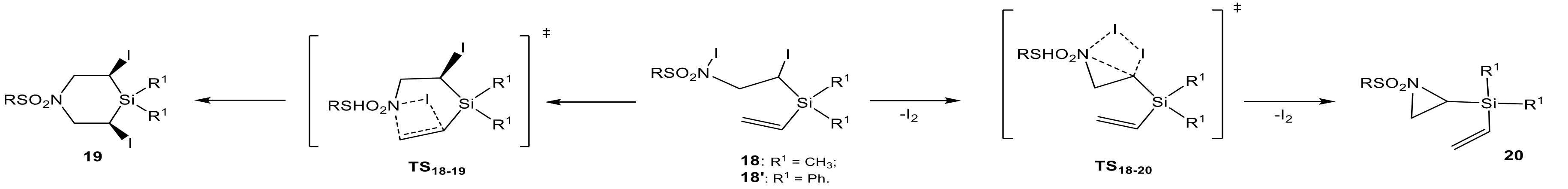

R = CH_3 (**a**), CF_3 (**b**), *p*-Tol (**c**), *p*-Nos (**d**).

								**R^1 = CH_3**									
**Parameter**	**18**	**$TS_{18\text{-}19}$**				**$TS_{18\text{-}20}$**				**19***				**20**			
		**a**	**b**	**c**	**d**	**a**	**b**	**c**	**D**	**a**	**b**	**c**	**d**	**a**	**b**	**c**	**d**
								MP2/B3LYP									
ΔE+ZPVE	0.0	34.2	**	28.3	33.2	7.9	***	10.5	7.8	–41.6	–44.4	–40.7	–40.7	2.7	3.1	4.0	3.9
ΔG	0.0	36.3	**	30.1	34.4	20.9	***	24.0	21.1	–40.0	–42.5	–39.0	–39.7	–7.7	–7.2	–6.9	–6.9
								wB97XD									
ΔE+ZPVE	0.0	46.7	**	47.7	47.6	6.1	***	–	–	–42.4	–44.2	–41.3	–41.3	–2.8	–2.1	–3.3	–3.9
ΔG	0.0	49.1	**	50.7	50.4	19.8	***	–	–	–40.1	–42.8	–38.7	–39.0	–13.0	–12.9	–12.3	–13.4
								R^1 = Ph									
Parameter	**18′**	**$TS_{18′\text{-}19′}$**				**$TS_{18′\text{-}20′}$**				**19′***				**20′**			
		**a**	**b**	**c**	**d**	**a**	**b**	**c**	**d**	**a**	**b**	**c**	**d**	**a**	**b**	**c**	**d**
								MP2/B3LYP									
ΔE+ZPVE	0.0	34.1	50.3	33.6	32.7	6.1	***	10.3	7.5	–41.2	–44.1	–40.5	–40.7	2.4	2.7	4.3	4.1
ΔG	0.0	35.3	52.9	34.8	35.8	19.8	***	23.5	21.2	–38.9	–41.7	–37.8	–37.2	–8.8	–8.1	–7.1	–5.4
								wB97XD									
ΔE+ZPVE	0.0	45.6	57.2	45.7	46.4	5.4	***	–	–	–42.1	–43.5	–41.3	–41.3	–3.3	–2.7	–1.8	–2.3
ΔG	0.0	48.0	59.5	50.4	48.9	18.0	***	–	–	–39.8	–41.5	–37.7	–39.4	–14.3	–14.4	–11.9	–13.1

* *Chair* conformation.

** Spontaneous cyclization with synchronous elimination of CF_3SO_2 occurred.

*** Not located.
